# The Effect of Bariatric Surgery on Healthcare Costs and Labor Market Attachment

**DOI:** 10.1007/s11695-022-05913-4

**Published:** 2022-01-26

**Authors:** Mette Bøgelund, Nils B. Jørgensen, Sten Madsbad, Maria Spanggaard, Ulrik H. Panton, Mikkel H. Pedersen, Pierre Johansen

**Affiliations:** 1 Incentive, Holte Stationsvej 14, 1, 2840 Holte, Denmark; 2 Department of Endocrinology, Hvidovre Hospital, Kettegaard Allé 30, 2650 Hvidovre, Denmark; 3 Novo Nordisk North West Europe Pharmaceuticals A/S, Ørestads Boulevard 108, 2300 København S, Denmark

**Keywords:** Bariatric surgery, Burden of disease, Cost of illness, Obesity

## Abstract

**Purpose:**

We aimed to estimate the total cost of bariatric surgery in Denmark.

**Materials and Methods:**

The study population included all Danish citizens ≥ 18 years who had received bariatric surgery, identified in the Danish National Patient Register in the period from 2002 to 2018. Patients who had received bariatric surgery were matched with three controls on gender, year of birth, and region of residence. A difference-in-difference approach was used to estimate the healthcare costs attributable to bariatric surgery from 3 years before to 5 years after surgery.

**Results:**

Total healthcare costs for cases receiving bariatric surgery during the first 5 years following surgery amounted to EUR 32,899, and EUR 16,651 for their matched controls. Thereby, the difference in total healthcare costs (EUR 16,248) between persons receiving bariatric surgery and their matched controls was 2.2 times the DRG rate for the surgery itself (EUR 7387).

Moreover, the results suggest that receiving bariatric surgery led to a total increase in gross earnings of EUR 5970 (5%) and a total reduction in receipt of transfer payments of EUR 4488 (12%) in the period up until 5 years after surgery.

**Conclusion:**

The results showed a significant and persistent increase in healthcare costs for people with obesity receiving bariatric surgery during the first 5 years after surgery. We also found that bariatric surgery was associated with increased attachment to the labor market.

**Graphical abstract:**

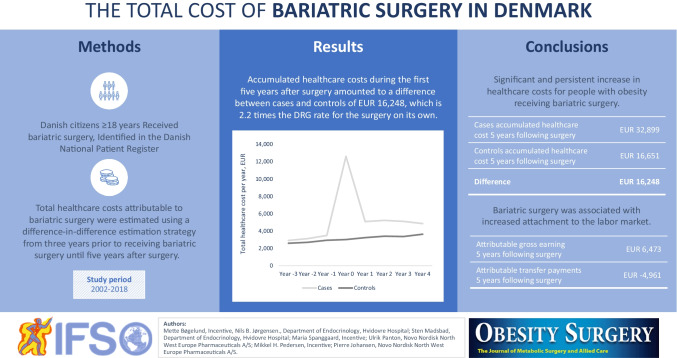

**Supplementary Information:**

The online version contains supplementary material available at 10.1007/s11695-022-05913-4.

## Introduction

It is a well-established fact that the prevalence of obesity has increased substantially during the past decades [1]. In Denmark, the 2017 obesity rate was estimated to be 17% of the adult population [2]. Moreover, it has been estimated that the prevalence of extreme obesity (BMI > 40 > kg/m^2^) has risen exponentially from 0.1 to 0.8% for men and from 0.4 to 1.5% for women in the period from 1985 to 2014.

Severe and extreme obesity have been shown to be associated with increased morbidity and mortality [3, 4]. Pharmacological treatment of patients with anti-obesity medication (AOM) is only applied in clinical practice for the treatment of obesity to a limited extent, and there are few available treatments with only minor or moderate effect on body weight [5].

In recent decades, bariatric surgery has become an increasingly popular surgical procedure.

Bariatric surgery has been shown to cause a sustained weight loss of about 40 kg or 15 BMI units and reduction in comorbidities and mortality [6, 7].

If the established effects of bariatric surgery on weight loss and reduction in comorbidities translate to effects on labor market attachment and earnings, it becomes highly relevant to establish both the total healthcare costs and the attributable effects on earnings and receipt of transfer payments (defined as social benefits primarily paid by the state such as pensions, sick leave, early retirement payments, and unemployment benefits) to assess the true cost-effectiveness of the procedure. It is also a well-established fact that bariatric surgery has a high risk of long- and short-term complications. For that reason, it becomes relevant to estimate the total healthcare and indirect costs as income and transfer payment associated with bariatric surgery.

In 2008, Larsen and co-authors [8] found that bariatric surgery was associated with a statistically non-significant increase in healthcare costs, which was mostly attributed to inpatient hospitalizations. In addition to this, the study found a significant decrease in use of prescription medicine, as well as a slight positive effect on receipt of transfer payments and no significant effect on income.

Due to the study designs, previous epidemiologic and health economic research have been limited to estimating correlations between bariatric surgery and the endpoints of interest. In this study, we aim to estimate the direct and indirect costs associated with bariatric surgery. We suggest an alternative definition of the control group, using patients who undergo bariatric surgery 6 years later. This was done to increase comparability between the controls and the cases and remove the bias originating from differences in disease characteristics as well as unobservable differences between the groups.

## Material and Methods

### Study Population

We performed a register-based retrospective cohort study which included adult patients who received bariatric surgery at the age of ≥ 18 at the date of bariatric surgery, i.e., the first registration with the procedure code KJDF (including sublevels). To ensure that the estimated healthcare costs in the year of surgery captured all costs related to bariatric surgery, we defined the index date as the date 3 months before the date of surgery.

### Matching with Control Population

Each case was matched with three controls who received bariatric surgery 6 years after the case. We restricted the study period from 3 years prior to the case’s index date to 5 years after, which meant that controls were only observed in the period leading up to their bariatric surgery, i.e., from 9 years before to 1 year before surgery. This ensured similarity in disease characteristics between cases and controls. Then, cases and controls were exactly matched on gender and region of residence and matched on date of birth using nearest neighbor matching. An illustration of the matching procedure is presented in the supplementary material along with an illustration in Suppl. Figure 1. This approach has previously been used to evaluate the effect of non-randomly assigned interventions [9, 10].

#### Data Sources

All Danish residents are assigned a personal identification number, which is recorded in the Central Person Register along with information on date of birth, gender, and family relationships [11]. The personal identification number enables secure linkage of individuals across Danish national registers.

Patient-level data on somatic hospitalization was retrieved from the Danish National Patient Register (DNPR), which holds information on all somatic hospitalizations, outpatient activities, and emergency room contacts [12]. Data on operational procedures are recorded in the DNPR as procedure codes. Data on costs for healthcare services performed at Danish hospitals are registered separately as diagnosis-related group (DRG) tariffs and were taken from the DRG registers. Contacts and costs in primary care are recorded in the National Health Service Register [13]. Information on use of prescription medicines sold at Danish pharmacies was collected from the Danish National Prescription Register [14]. Finally, information on gross earnings and transfer payments was taken from the Danish Income Register [15].

#### Endpoints

Total healthcare costs included cost of inpatient hospitalizations, outpatient visits, psychiatric hospital contacts, and cost of prescription medicines and fees for general practitioners and specialists, which were priced using the DRG tariffs for admissions and outpatient visits, according to the DNPR.

Indirect costs as measured by productivity loss were proxied by earnings^1^ and receipt of transfer payments.

Finally, we estimated healthcare utilization as the average number of inpatient hospitalizations and outpatient contacts per year.

#### Data Analysis

The study population was followed from 3 years prior to index date (date of surgery) and up to 5 years after index date, death, emigration, or 31 December 2018, whichever occurred first.

The effect of bariatric surgery on each defined endpoint was evaluated using two separate approaches: actual costs and attributable costs.

Actual costs were estimated as the cases’ and controls’ average costs in each year relative to the date of surgery for the case.

The cost and healthcare resource use attributable to bariatric surgery were estimated using a regression-based (ordinary least squares) difference-in-difference estimation strategy. In essence, this meant comparing the difference in the outcomes between cases and controls in each year with the difference in the year before the case received surgery.

In subgroup analyses, the direct and indirect costs were estimated according to the type of surgery, gender, age category, and year of surgery.

#### Ethical Considerations

The study was register-based and complied with the regulations set up by the Danish Data Protection Agency (J. nr. 2014–54-0664). No ethical approval was needed.

## Results

### Population

A total of 19,826 persons received bariatric surgery between 1997 and 2018, according to the DNPR, of whom we were able to find suitable matches for 14,009 persons, who then comprised the study population. Among the study population, 77% were female and the average age at index date was 40.1 (SD: 9.7) years (Table 1). Eighty-three percent of the cases received bariatric surgery before the change in the eligibility criteria in 2010.^2^ Finally, 92% of persons included in the case population received gastric bypass.

**Table 1:** Characteristics of the study population

	Cases, *N* (%)	Controls, *N* (%)
Total	14,009	40,298
Gender		
Men	3,155 (23%)	8,968 (22%)
Women	10,854 (77%)	31,330 (78%)
Age at surgery		
Mean age (*SD*)	40.07 (9.65)	39.79 (9.2)
18–24 years	720 (5%)	1,993 (5%)
25–34 years	3,444 (25%)	10,093 (25%)
35–44 years	5,297 (38%)	15,503 (38%)
45–55 years	3,411 (24%)	10,643 (26%)
Above 55 years	1,137 (8%)	2,066 (5%)
Index year		
2002–2010	11,599 (83%)	33,382 (83%)
2011–2018	2,410 (17%)	6,916 (17%)
Type of surgery		
Gastric bypass	12,951 (92%)	NA
Gastric banding	957 (7%)	NA
Other	101 (1%)	NA
Educational level, *n* (%)		
Primary or no education	4,058 (29%)	11,368 (28%)
Secondary	6,668 (48%)	18,489 (46%)
Short cycle tertiary	508 (4%)	1,594 (4%)
Bachelor’s or equivalent	2,302 (16%)	7,010 (17%)
Master’s or higher	259 (2%)	998 (2%)
Education unknown	214 (2%)	839 (2%)
Employment status in index year		
Employed	9,564 (68%)	28,875 (72%)
Unemployed	4,445 (32%)	11,423 (28%)

The table presents the characteristics of the cases in the study population and disease characteristics of the persons who received bariatric surgery, included in the study population in the index year

Abbreviations: *SD* standard deviation, *n* number of persons

### Healthcare Costs

The yearly healthcare costs for cases receiving bariatric surgery and their matched controls in the 3 years up to the index date were statistically significant different, with slightly higher costs in the operated population.

The average yearly individual healthcare costs throughout all post-surgery years were also estimated to be statistically significant different and more than 97% higher among persons who received bariatric surgery compared with their matched controls.

Total yearly healthcare costs were the highest in the year following the index date, with an estimated increase of EUR 9046 (*p* < 0.01), of which the direct cost of the procedure made up an average of EUR 7387. In the years after surgery (year 1 to year 4), bariatric surgery was estimated to increase the yearly healthcare costs significantly, between EUR 1303 (*p* < 0.01) in year 1 to EUR 673 (*p* < 0.01) in year 4. The difference-in-difference estimates are presented in the top panel of Table 2.

**Table 2:** Attributable costs of bariatric surgery per year 3 years prior to surgery to 5 years after, EUR per person

	Year -3	Year -2	Year -1 (base year)	Year 0	Year 1	Year 2	Year 3	Year 4	Total(Years0-4)
Total population (*n* = 14,009)									
Cost of inpatient hospitalizations	197^***^	194^***^	0	8,902^***^	1,653^***^	1,808^***^	1,844^***^	1,426^***^	15,634
Cost of somatic inpatient hospitalizations	26	180^***^	0	8,808^***^	1,452^***^	1,570^***^	1,584^***^	1,138^***^	14,553
Cost of psychiatry inpatient hospitalizations	171^***^	14	0	94^***^	201^***^	238^***^	262^***^	287^***^	1,082
Cost of outpatient visits	− 404^***^	− 308^***^	0	290^***^	− 154^***^	− 305^***^	− 420^***^	− 481^***^	− 1,070
Cost of somatic outpatient contacts	− 442^***^	− 361^***^	0	299^***^	− 178^***^	− 343^***^	− 468^***^	− 537^***^	− 1,228
Cost of psychiatry outpatient contacts	38^**^	53^***^	0	− 9	24	37^**^	49^***^	57^***^	158
Primary care visits	− 12^**^	− 14^**^	0	− 6	− 33^***^	− 49^***^	− 60^***^	− 73^***^	− 220
Prescription medicine	− 10	6	0	− 140^***^	− 164^***^	− 179^***^	− 169^***^	− 200^***^	− 851
Anti-obesity medication	2	1	0	− 6^***^	− 12^***^	− 11^***^	− 9^***^	− 9^***^	− 47
Psycholeptics and psychoanaleptics	− 2	2	0	− 15^***^	− 14^***^	− 7^*^	0	6	− 30
Other prescription medicine	− 10	3	0	− 119^***^	− 138^***^	− 162^***^	− 159^***^	− 196^***^	− 774
*Total attributable healthcare costs*	− 229^***^	− 123	0	9,046^***^	1,303^***^	1,275^***^	1,195^***^	673^***^	13,493
Patients receiving gastric bypass (*n* = 12,951)									
Cost of inpatient hospitalizations	243^***^	246^***^	0	8,665^***^	1,741^***^	1,923^***^	1,945^***^	1,524^***^	15,800
Cost of outpatient visits	− 385^***^	− 295^***^	0	252^***^	− 159^***^	− 281^***^	− 409^***^	− 454^***^	− 1,051
Primary care visits	− 11^*^	− 15^*^	0	− 5	− 32^***^	− 47^***^	− 61^***^	− 72^***^	− 218
Prescription medicine	− 8	6	0	− 143^***^	− 164^***^	− 183^***^	− 171^***^	− 202^***^	− 864
*Total attributable healthcare costs*	− 160^*^	− 57	0	8,768^***^	1,387^***^	1,412^***^	1,303^***^	796^***^	13,666
Patients receiving other type of surgery (*n* = 1,058)									
Cost of inpatient hospitalizations	− 320	− 445	0	11,779^***^	539^*^	360	568^*^	189	13,435
Cost of outpatient visits	− 601^***^	− 459^***^	0	746^***^	− 115	− 622^***^	− 561^***^	− 820^***^	− 1,371
Primary care visits	− 27	− 9	0	− 17	− 43	− 66^**^	− 48^*^	− 84^***^	− 259
Prescription medicine	− 35	1	0	− 109^**^	− 155^***^	− 137^***^	− 136^***^	− 164^***^	− 701
*Total attributable healthcare costs*	− 983^***^	− 911^**^	0	12,400^***^	227	− 466	− 177	− 879	11,105

The table presents the difference-in-difference estimates for patients receiving bariatric surgery compared with their matched controls by cost category. Estimates were significant at *10%, **5%, ***1%

**Fig. 1:**
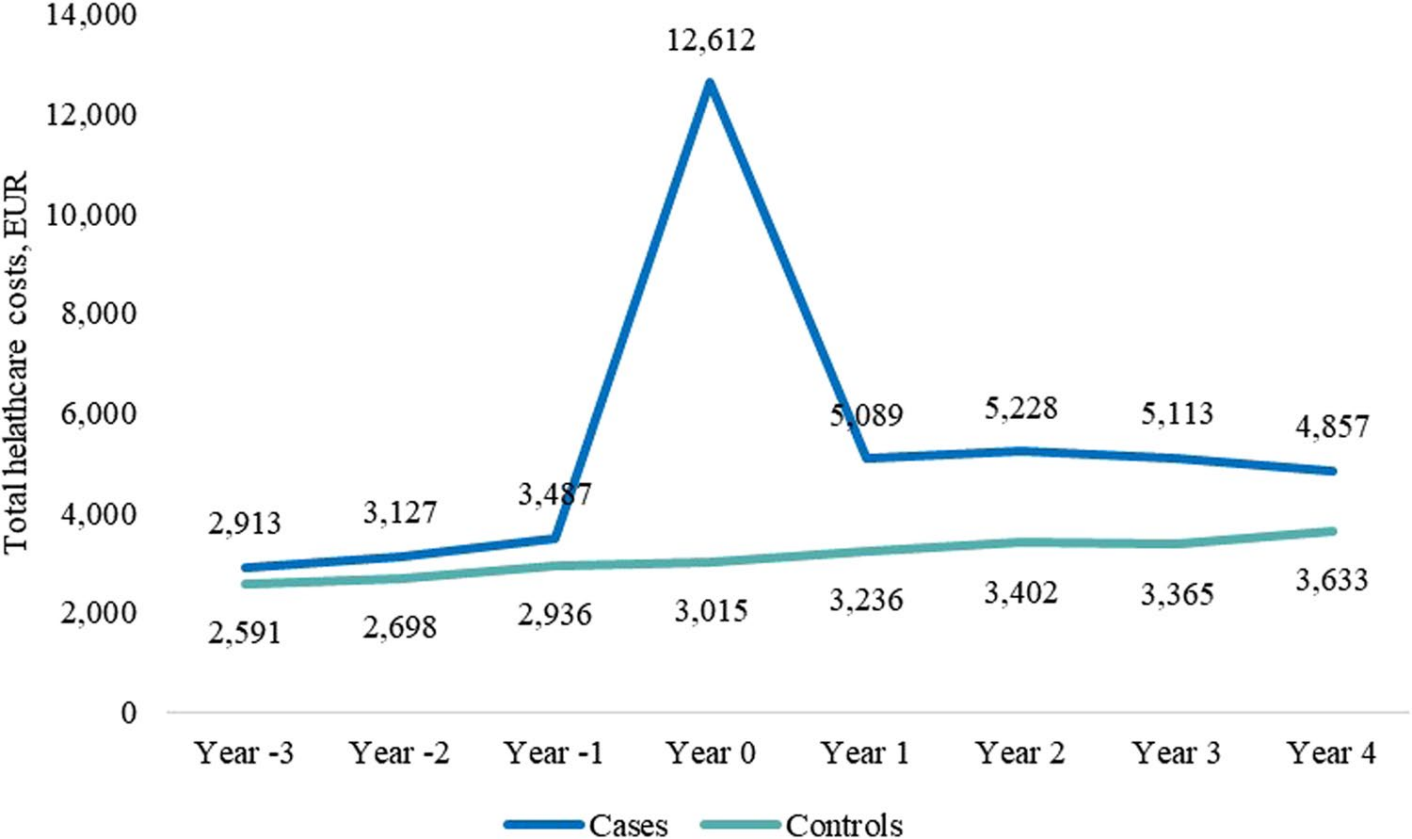
Total healthcare costs per year relative to receiving bariatric surgery for cases and controls, EUR

The mean total healthcare costs per year for persons receiving bariatric surgery for cases and their matched controls are presented in Fig. 1. The figure shows that accumulated healthcare costs during the first 5 years after surgery amounted to EUR 32,899 for cases receiving bariatric surgery and EUR 16,651 for their matched controls. This implied a difference of EUR 16,248, which is 2.2 times the DRG rate for the surgery on its own (EUR 7387).

**Fig. 2:**
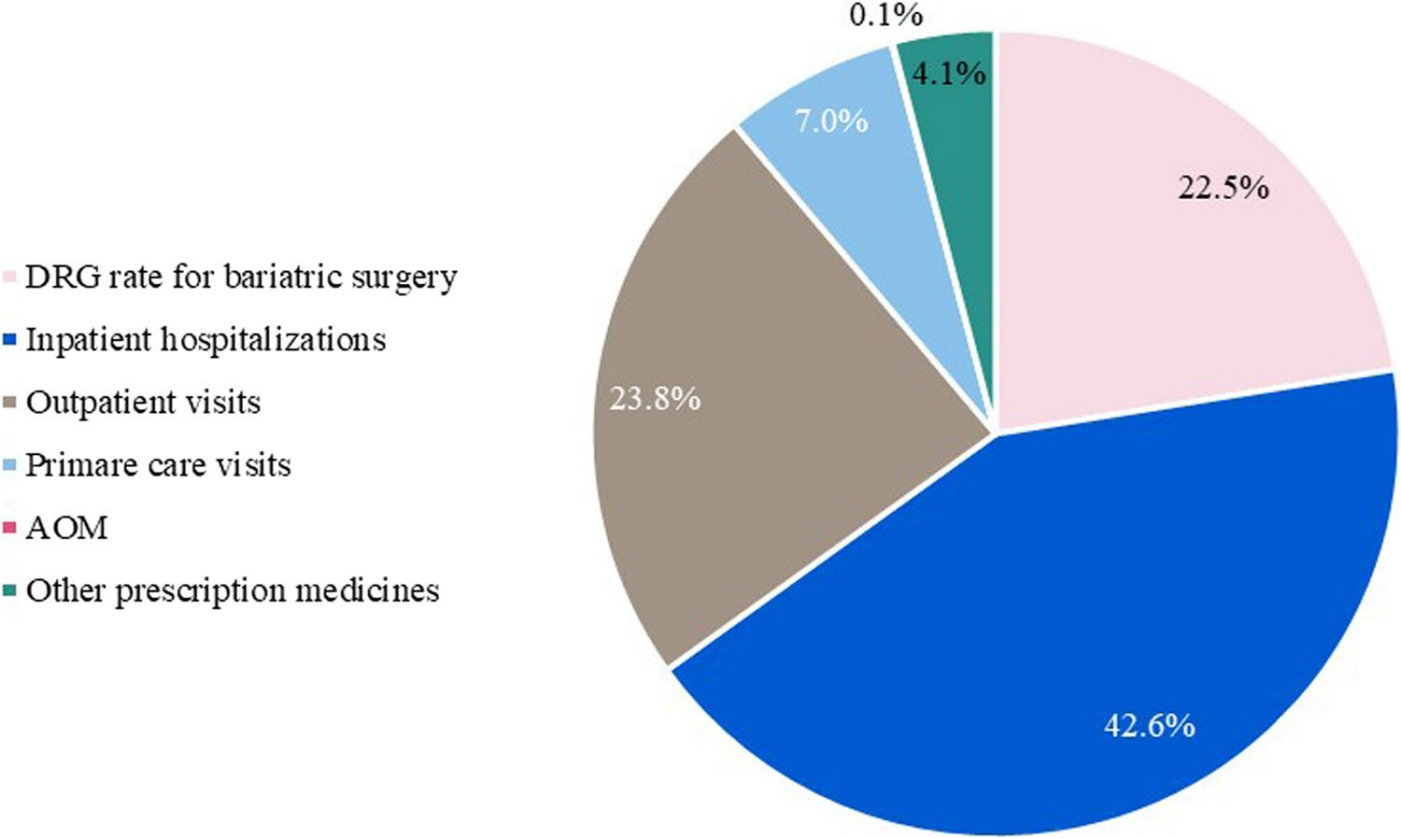
Breakdown of total healthcare costs among cases from year 0 to year 4 and b attributable healthcare costs from year 0 to year 4. Abbreviations: AOM = Anti-obesity medication, DRG = Diagnosis-related group.

Figure 2 illustrates the breakdown of  total health care cost among cases. The main driver of the increase in individual healthcare costs in the year after surgery was identified to be costs related to somatic inpatient hospitalizations. Even so, bariatric surgery was estimated to reduce healthcare costs associated with outpatient visits as well as costs related to primary care visits and prescription medicines. It is important to note that the total attributable healthcare costs per year gradually decreased and were EUR 673 at year 4 after surgery.

The remainder of Table 2 presents the estimates of healthcare costs attributable to bariatric surgery per year from 3 years before surgery to 4 years after surgery by type of gastric procedure. Overall, results showed similar patterns for persons who had received gastric bypass and for persons who had received gastric banding or sleeve gastrectomy.

### Effect on Gross Earnings and Transfer Payments

Bariatric surgery influenced both earnings and receipt of transfer payments as shown in Table 3. Specifically, no statistically significant changes in earnings and receipt of transfer payments were observed in the year of surgery. The opposite was detected for both outcomes in the period after surgery, where earnings increased significantly and the receipt of transfer payments decreased significantly. In total, the estimates suggest that bariatric surgery increased total earnings per person by EUR 5970 and reduced total receipt of transfer payments per person by EUR 4488 from the year of surgery until 4 years after surgery. In addition to this, we found that on average 45.7% of persons receiving bariatric surgery increased their yearly earnings in the post-surgery years and 41.7% reduced their receipt of transfer payments.

**Table 3:** Attributable earnings and receipt of transfer payments per year 3 years prior to surgery to 5 years after, EUR per person

	Year -3	Year -2	Year -1 (base year)	Year 0	Year 1	Year 2	Year 3	Year 4	Total cost (years 0–4)
All patients									
Gross earnings	171	− 38	0	− 233	1,136^***^	1,178^***^	2,064^***^	1,826^***^	5,970
Receipt of transfer payments	− 225	− 228	0	114	− 682^***^	− 1,265^***^	− 1,270^***^	− 1,384^***^	− 4,488
Patients receiving bypass (*n* = 12,951)									
Gross earnings	96	− 48	0	− 153	1,230^***^	1,265^***^	2,174^***^	1,910^***^	6,473
Receipt of transfer payments	− 171	− 209	0	88	− 698^***^	− 1,284^***^	− 1,279^***^	− 1,409^***^	− 4,961
Patients not receiving bypass (*n* = 1,058)									
Gross earnings	1,050	43	0	− 1,171	17	129	740	801	516
Receipt of transfer payments	− 836	− 421	0	382	− 525	− 1,061^*^	− 1,193^**^	− 1,098^*^	− 3,495

The table presents the difference-in-difference estimates for gross earnings and receipt of transfer payments for patients receiving bariatric surgery compared with their matched controls by cost category and type of surgery. Estimates were significant at *10%, **5%, ***1%

Again, the estimates suggest that the effect of receiving bariatric surgery on a person’s labor market attachment, shown in gross earnings and receipt of transfer payments, was similar between persons receiving gastric bypass and persons receiving bariatric surgery in the form of sleeve gastrectomy or gastric banding, although the estimated effect was significant and slightly stronger for patients receiving gastric bypass.

### Subgroup Analysis

Table 4 presents the estimates of the total attributable healthcare costs, earnings, and receipt of transfer payments from the year of surgery until 4 years after surgery for different subgroups. The subgroup analyses showed that women receiving bariatric surgery had higher healthcare costs in the years following surgery, EUR 14,980, relative to men, EUR 8341, and also experienced a higher increase in earnings.

**Table 4:** Total attributable healthcare costs, earnings, and receipt of transfer payments by subpopulation from year 0 to year 4, EUR per person

	Total healthcare costs	Total earnings	Total transfer payments
Gender			
Male	8,341	4,565	− 4,594
Female	14,980	6,372	− 4,459
Age-group			
Age 18–24	17,798	11,322	− 7,161
Age 25–34	15,663	8,661	− 7,496
Age 35–44	13,705	7,045	− 3,263
Age 45–54	10,616	2,642	− 3,689
Age 55 +	11,780	− 749	− 1,742
Period of surgery			
Surgery before 2010	14,361	8,907	− 5,613
Surgery after 2010	12,529	2,717	− 3,246

The table presents total attributable healthcare costs, earnings, and receipt of transfer payments by subpopulation estimated as the sum of the difference-in-difference estimates for each outcome in the period from index date to 4 years after surgery

In addition to this, the subgroup analyses showed higher healthcare costs, higher increase in earnings, and reduction in receipt of transfer payments for persons receiving bariatric surgery at a young age.

## Discussion

This study documented that there were substantial healthcare costs connected to receiving bariatric surgery up to 5 years after surgery. Total attributable healthcare costs in the 5 years following surgery were estimated to be EUR 13,493.

In addition to this, we found that receiving bariatric surgery increased the average labor market participation by statistically significantly increasing gross earnings by EUR 5970 and reducing receipt of transfer payments by EUR 4488 in the 5 years following surgery.

These results contradict the findings of Larsen and co-authors [8], who were unable to detect significant differences in healthcare costs, except in the year of surgery, when comparing patients who received bariatric surgery in 2010 with a matched control group that contained persons who did not undergo bariatric surgery but did meet the criteria for bariatric surgery. Similarly, the study was not able to detect significant effects of receiving bariatric surgery on income and transfer payments during the first 3 years after surgery.

We ascribe the differences in the results to the difference in the choice of a control group that enables us to make a causal interpretation of the estimates from non-experimental data by ensuring similarity in disease characteristics between cases and controls.

The results highlight that the costs attributable to bariatric surgery are much higher than the DRG rate for the surgery itself and thereby emphasize the importance of employing robust cost estimates when modeling the cost-effectiveness of interventions in the treatment of obesity.

Although substantial costs are connected to bariatric surgery, the procedure leads to an improvement in health and quality of life among this group of people which is also evident in the increased labor market attachment after bariatric surgery.

### Limitations

One potentially confounding factor of the study is caused by the definition of the control group. By matching patients who receive bariatric surgery with controls who also receive surgery but 6 years later, the cases and the controls will potentially not be at the same disease stage during the study period. In addition to this, it also meant that we were only able to include patients who received surgery before 2013 in the case population.

Finally, the eligibility criteria for receiving bariatric surgery were changed in 2010, and the current analysis did not account for this in the matching procedure, and therefore, the analysis will in some cases compare the outcomes of a person receiving bariatric surgery because they fulfilled the eligibility criteria prior to 2010 with the outcomes of persons who would later receive bariatric surgery because they fulfilled the eligibility criteria after 2010. However, it is important to note that the criteria were narrowed down by increasing the BMI for fulfilling the criteria for bariatric surgery, and therefore, this could lead us to underestimate the total healthcare costs in the outlined scenario.

Additionally, it is important to note that only very few sleeve gastrectomies were performed in Denmark before 2016. Therefore, our study does not allow us to conclude anything with respect to the costs associated with gastric sleeve surgery.

The design of the study did not allow for longer follow-up, but as total healthcare costs gradually declined with time from surgery, longer observation time after surgery could yield other results.

## Conclusion

The results showed a significant increase in healthcare costs for people with obesity who received bariatric surgery during the first 5 years after surgery. The difference in total healthcare costs was estimated to be 2.2 times the DRG rate for the surgery itself, even though year-over-year total healthcare costs declined with time from surgery. We also found that bariatric surgery was associated with increased attachment to the labor market.

Supplementary Information.

## Supplementary Information

Below is the link to the electronic supplementary material.

Supplementary file1 (DOCX 101 KB)

## Footnotes

1 Gross earnings consist of salary (including taxable and non-taxable salary, fringe benefits, severance payments, value of stock options, and sickness benefits paid by the employer), net profits from self-employed business, and other taxable income (including fees from lectures and consulting business).

2 Before 2010, persons aged 18 and above were eligible for receiving bariatric surgery in Denmark if they had a BMI > 40 or had a BMI > 35 and were diagnosed with at least one comorbidity. From December 2010, the eligibility criteria were restricted such that it was only persons with a BMI > 50 and persons with a BMI > 40 who had at least one comorbidity who were eligible for receiving bariatric surgery.
