# Supplementary material for: The Effect of Bariatric Surgery on Healthcare Costs and Labor Market Attachment
Source: Obes Surg. 2022 Jan 26;32(4):998–1004. doi: 10.1007/s11695-022-05913-4 (PMC8933378; doi:10.1007/s11695-022-05913-4)
Supplement: Supplementary file 1 — Supplementary file1 (DOCX 101 KB) [file 11695_2022_5913_MOESM1_ESM.docx]

Supplementary material: The effect of bariatric surgery on healthcare costs and labor market attachment

**Authors and affiliation:**Mette Bøgelund, Incentive, Holte Stationsvej 14, 1, 2840 Holte, Denmark

Nils B. Jørgensen., Department of Endocrinology, Hvidovre Hospital, Kettegaard Allé 30, 2650 Hvidovre, Denmark

Sten Madsbad, Department of Endocrinology, Hvidovre Hospital, Kettegaard Allé 30, 2650 Hvidovre, Denmark

Maria Spanggaard, Incentive, Holte Stationsvej 14, 1, 2840 Holte, Denmark

Ulrik Panton, Novo Nordisk North West Europe Pharmaceuticals A/S, Ørestads Boulevard 108, 2300 København S, Denmark

Mikkel H. Pedersen, Incentive, Holte Stationsvej 14, 1, 2840 Holte, Denmark

Pierre Johansen, Novo Nordisk North West Europe Pharmaceuticals A/S, Ørestads Boulevard 108, 2300 København S, Denmark

Suppl. Figure 1: Illustration of the matching procedure

**Step 1**


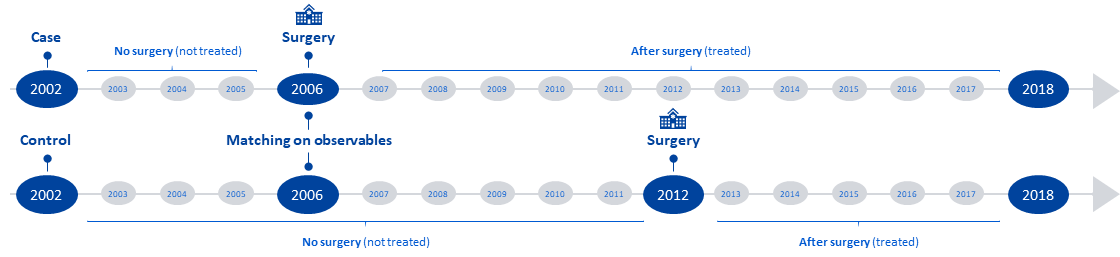


**Step 2**
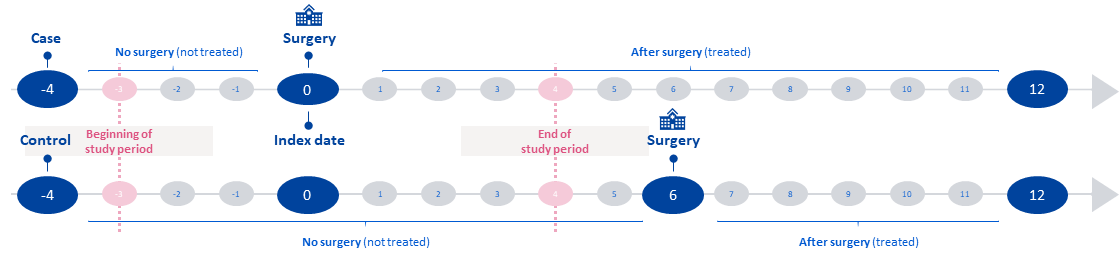


**Step 3**


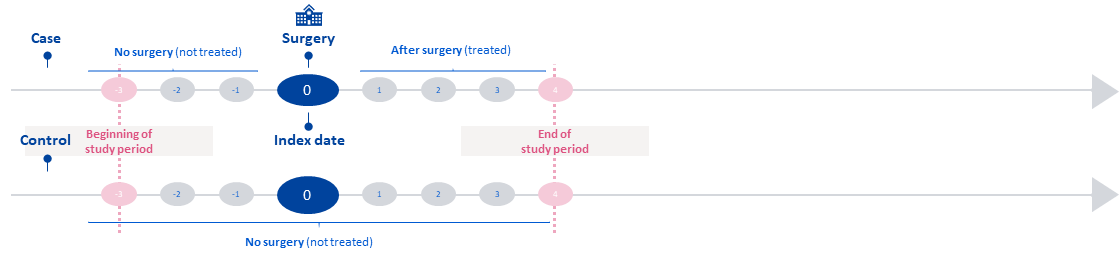


The figure illustrates an example of the three-step procedure for matching cases and controls applied in the study. In step one, the figure presents the timeline (2002 to 2018) of where we have data available for the cases (top) and the controls (bottom). In this example, the case receives surgery in 2006, and the control receives surgery in 2012 (in the matching procedure, we use the exact date of surgery, but for simplicity, we use year of surgery in this example).

In the next step, we matched the case with three of the identified controls on gender, birth date and region of residence in year 2006, the year in which the case received surgery. Hereafter, we aligned the years so that they represent the year relative to the date of surgery for the case.

In our example, this implies that we observe the case for four years prior to surgery (year -4 to -1) and 13 years after surgery (year 0 to 12).
Similarly, we observe the controls for 10 years prior to surgery (year -4 to 5) and seven years after surgery (year 6 to 12).

Lastly, in step three, we restricted the study period to the years -3 to 4 for both the case and the controls. Thereby, we observed the case for three years prior to surgery and five years after surgery and, more importantly, only observed the controls in the period prior to surgery.
